# Urban–Rural Differences in School Districts’ Local Wellness Policies and Policy Implementation Environments

**DOI:** 10.3390/nu16060801

**Published:** 2024-03-11

**Authors:** Swati Iyer, Timothy J. Walker, Alexandra L. MacMillan Uribe, Chad D. Rethorst, Rebecca A. Seguin-Fowler, Jacob Szeszulski

**Affiliations:** 1 School of Medicine, Texas A&M University, 8447 Riverside Pkwy, Bryan, TX 77807, USA; 2 Department of Health Promotion & Behavioral Sciences, Center for Health Promotion and Prevention Research, The University of Texas Health Science Center at Houston School of Public Health, 7000 Fannin St., Houston, TX 77030, USA; 3 Texas A&M Institute for Advancing Health through Agriculture (IHA), 17360 Coit Rd, Dallas, TX 75252, USA; 4 Texas A&M Institute for Advancing Health through Agriculture (IHA), 1500 Research Parkway, Centeq Building B, College Station, TX 77845, USA

**Keywords:** physical activity, exercise, nutrition, children, adolescent

## Abstract

Higher rates of obesity in rural compared to urban districts suggest environmental differences that affect student health. This study examined urban–rural differences in districts’ local wellness policies (LWPs) and LWP implementation environments. Cross-sectional data from two assessments in Texas were analyzed. In assessment one, each district’s LWP was reviewed to see if 16 goals were included. In assessment two, an audit was conducted to identify the presence of a wellness plan (a document with recommendations for implementing LWPs), triennial LWP assessment, and school health advisory councils (SHACs) on the district website. Rural districts’ LWPs had a smaller number of total goals (B = −2.281, *p* = 0.014), nutrition education goals (B = −0.654, *p* = 0.005), and other school-based activity goals (B = −0.675, *p* = 0.001) in their LWPs, compared to urban districts. Rural districts also had lower odds of having a wellness plan (OR = 0.520, 95% CI = 0.288–0.939), *p* = 0.030) and a SHAC (OR = 0.201, 95% CI = 0.113–0.357, *p* < 0.001) to support LWP implementation, compared to urban districts. More resources may be needed to create effective SHACs that can help develop and implement LWPs in rural areas. Important urban–rural differences exist in Texas LWPs and LWP implementation environments.

## 1. Introduction

United States children and adolescents continue to be affected by increasingly unhealthy lifestyles that predispose them to multiple chronic diseases (e.g., cancer, cardiovascular disease, or metabolic syndrome) [1,2]. Obesity, physical activity, and diet are modifiable risk factors for these chronic diseases, and children with obesity are twenty times more likely than healthy weighted children to have obesity as adults [3,4]. Furthermore, there is a disproportionately higher rate of obesity for children living in rural communities compared to children living in urban communities [5]. Thus, it is important to set guidelines, improve environments, and provide education that increases participation in healthy lifestyles in ways that can prevent urban–rural health disparities [6,7]. Improving engagement in preventive behaviors within rural communities can also decrease the number of youth developing chronic diseases that are attributable to lower levels of physical activity and poorer diet quality, as well as environmental differences that play a role in promoting or discouraging health behaviors [8,9,10].

Local wellness policies (LWP)—district-level policies about physical activity and nutrition that are required for all districts that participate in federal school meal programs—are one avenue for providing children with effective preventive measures for their health, as they can improve school district health environments, support educational initiatives, and in turn, improve students’ physical activity and eating behaviors [11,12,13]. Children spend an average of 6.5 h in school per day [14,15], eat two meals per day at school [16], and, on average, receive up to 40% of their physical activity during school hours [17]. Comprehensive and well-implemented LWPs are consistently associated with superior school district health environments as well as nutritional and physical activity outcomes [18,19,20,21]. Thus, LWPs could play substantial roles in reducing childhood obesity [22,23]. High-quality LWPs can also help districts serve more nutritious meals, limit the opportunity and access to unhealthy snacks and overly sweetened drinks, and create a consistent structure for engaging students in physical activity [24]. Further, the inclusion of each additional wellness-based practice in an LWP is associated with a 3% reduction in the prevalence of obesity for students in that district [25]. Despite the many benefits of high-quality LWPs, it is unclear whether there are differences between urban and rural schools’ LWPs or what might be creating those differences.

LWPs have the potential to create an environment that can positively affect children’s health; however, LWPs cannot be effective if they are not well-written, implemented, and enforced [26,27,28]. In general, rural school districts face more challenges than urban school districts in implementing physical activity and nutrition programs, including fewer staff, a lack of financial and technical assistance, and greater difficulties procuring healthier foods [29,30]. Further, the barriers that rural districts face can be directly linked to differences in social determinants of health (e.g., infrastructure and capacity or community engagement) that are often found in rural communities [31,32]. It is unclear if these urban–rural differences in district and community health environments are also evident in schools’ LWP quality and implementation processes, including the creation of LWP plans (i.e., documents that provide guidance on processes for delivering LWPs), use of LWP assessments (required by federal law triennially), or in the availability of community stakeholders to review and make recommendations on LWP processes. Previous research has shown that some organizational supports, and in particular, school health advisory councils (SHACs), provide guidance for developing, implementing, evaluating, and monitoring LWPs [33]. 

The Whole School, Whole Community, Whole Child (WSCC) model emphasizes the importance of physical activity and nutrition within schools, and it includes 10 components (e.g., health education, community involvement, and family engagement) that are especially relevant for understanding school health environments [34]. These components have the potential to affect several aspects of school districts’ LWP processes, including development, implementation, and evaluation. Several strategies (i.e., audit and feedback and program champions) that incorporate aspects of the WSCC model have been shown to improve LWP implementation [27,35]. However, none of these strategies have been specifically designed or tailored for urban or rural school districts. A greater understanding of the differences between urban and rural districts’ health environments is needed to determine if and how strategies should be tailored.

Accordingly, the purposes of this study are to (1) understand the urban–rural differences in districts’ LWPs and (2) examine whether there are differences in environmental supports for implementing LWPs. We hypothesize that urban districts will have both more goals and support for implementing LWPs than rural districts. By better understanding the barriers rural schools face, districts can develop LWPs that are more tailored to their characteristics. Further, these findings may also help to develop implementation strategies (e.g., audit and feedback processes, policy champions) that can improve the implementation and enforcement of LWPs and, ultimately, improve students’ physical activity, nutrition, and obesity outcomes.

## 2. Materials and Methods

### 2.1. Participants 

We conducted a cross-sectional analysis using secondary data collected during two separate local needs assessments of LWPs from school districts around major urban centers in the state of Texas, USA. In the first needs assessment, our goal was to identify the types of goals that are written into districts’ LWPs. In the second needs assessment, our goal was to determine the type of tools and resources available to districts that support the implementation of their LWPs. This study does not involve human participants, and informed consent was therefore not required (The ethics statement is not applicable).

### 2.2. Needs Assessment #1

In the Spring of 2020, we selected one public health region in South Texas and all the counties in this area (*n* = 16). Using the Texas Education Agency’s school locator, we identified all public school districts within this region, excluding charter schools, because they are not always required to develop LWPs (N = 117; 1784 schools; 1,440,580 students). For each school district, we used the Texas Association of School Boards (TASB) Policy Online Tool to collect LWP documents. If the LWP was not available via the online tool, we searched for the LWP on the school district’s website. Of the 117 available documents, 95 used the same template to develop their LWP, which we analyzed for this study. However, 16 LWPs used an outdated version of the template, and six independently developed LWPs (i.e., did not follow the template), which prevented us from making meaningful urban–rural comparisons. Thus, we excluded them from this analysis. Using the state LWP template as a coding guide, we reviewed the remaining 95 LWPs to determine whether each of the 16 goals that are listed in the state template across four content areas was included in the districts’ LWPs (Table 1). For more information on how the LWPs were coded, see Szeszulski et al., 2021 [36]. 

### 2.3. Needs Assessment #2

For the second needs assessment, we selected four education service regions in North Texas. Using the Texas Education Agency’s school locator, we identified all public school districts in those regions, excluding charter schools, because they are not always required to develop LWPs (N = 239; 2264 schools; 1,460,226 students). For each district, we conducted a district website audit to gauge how districts were implementing LWPs and their infrastructure for implementation. To do this, we identified specific pages on the website related to health and/or searched specific key terms, including the following: assessment, coordinated school health, health, health education, health services, nutrition, physical education, SHAC, school health advisory council, triennial assessment, wellness, wellness assessment, wellness plan, wellness policy, and wellness report card. We reviewed pages that were related to required policies, physical activity, and/or nutrition topics (e.g., coordinated school health, cafeteria, health and safety, health service, SHAC, accountability, and required postings). From the website, we identified if the district had a wellness plan (i.e., a document providing recommendations for implementing wellness policies), if they had completed their triennial assessment, and if they had a school health advisory committee (SHAC) in their district. In Texas, all districts are required to have a SHAC that supports the implementation of LWPs. We coded all webpages over a 1-month period in the Fall of 2021.

### 2.4. Demographic Characteristics

To gather each district’s descriptive information, we used the most recent publicly available Texas Education Agency data. Within this descriptive information, we collected the race/ethnicity makeup and proportion of the schools. We also identified the proportion of students who were eligible for free or reduced-price meals through the National School Lunch Program or Child Nutrition Program, the student/teacher ratio, and district revenue per student. Using data from the National Center for Education Statistics, we also recorded the location of the school as being urban (suburban or town) or rural. Finally, we calculated the number of months between 1 Jan 2021 and the date the SWP was issued.

### 2.5. Data Analysis

We conducted descriptive analysis for the two samples by calculating the means and standard deviations for sociodemographic characteristics. Next, we assessed the frequency of binary variables (rural vs. urban school districts, wellness plan, triennial assessment, and SHAC) and the normality of continuous variables using skewness and kurtosis statistics, normality tests (e.g., Kolmogorov–Smirnov), and a visual inspection of distributions (e.g., nutrition promotion goals, nutrition education goals, physical activity goals, school-based activities, and total physical activity and nutrition goals). 

We used a series of general linear regression models to examine the unadjusted and adjusted associations between the wellness policy goals (number of nutrition promotion goals, nutrition education goals, physical activity goals, school-based activities, and total physical activity and nutrition goals) and districts being located in a rural vs. urban setting. The covariates we included in the set of adjusted models were the percentage of students who were economically disadvantaged, the district’s revenue per 1000 students, and the average number of students per school. To determine the relation between support for implementing LWPs (the creation of a wellness plan, triennial assessment, and creation of a SHAC) and districts in a rural vs. urban setting, we used unadjusted and adjusted logistic regression models, adjusting for the same covariates as previously described. 

We performed all data analyses using SAS software, version 9.4, with a significance level of 0.05. We identified three outliers in the data that were affecting the normal distribution of the model residuals (total number of goals < 5). We conducted a sensitivity analysis with the outliers removed. However, the removal of the outliers in a sensitivity analysis did not affect the results. Thus, the results of the sensitivity analysis are not presented here, and the sample size remained unchanged.

## 3. Results

### 3.1. Study Sample

In samples 1 and 2, the proportion of districts that were rural was 42.1% and 37.7%, respectively, which were not significantly different from one another (Table 2). However, sample 1 had more students per school, students per district, and students per teacher, whereas sample 2 had more revenue per student. The percentage of students that qualified as economically disadvantaged was also similar across the samples; however, all racial and ethnic demographic characteristics were different between the samples, as sample 1 had more students that identified as Black and Hispanic, whereas sample 2 had more students that identified as White.

### 3.2. Wellness Policy Goals

When analyzing the urban–rural differences in number of LWP goals (Table 3), we found that rural districts’ LWPs had a significantly smaller number of total goals (B = −2.35, *p* = 0.003), nutrition promotion goals (B = −0.316, *p* = 0.034), nutrition education goals (B = −0.536, *p* = 0.006), physical activity goals (B = −0.830; *p* = 0.038), and other school-based goals (B = −0.668, *p* < 0.001) compared to urban districts’ LWPs in the bivariate model. For the nutrition promotion goals and physical activity goals, this difference was no longer statistically significant when the model was adjusted for covariates (the socioeconomic status of students, the district’s revenue, and the number of students per school). However, the revenue per student (B = −0.246, *p* = 0.048) was significantly related to the number of physical activity goals. In the adjusted model, the total number of goals (B = −2.281, *p* = 0.014), nutrition education goals (B = −0.654, *p* = 0.005), and other school-based activity goals (B = −0.675, *p* = 0.001) remained significantly lower in rural districts compared to urban districts.

### 3.3. Supports for Implementing Local Wellness Policies

Rural districts had about 48% lower odds of having a wellness plan (23.3% vs. 36.9%, *p* = 0.030) and 80% lower odds of having a SHAC (43.3% vs. 79.2%, *p* < 0.001) compared to urban districts in the unadjusted model. However, these differences were not statistically significant in the adjusted models (Table 4). In the adjusted model, each thousand dollars in revenue per student (OR = 0.827, 95% CI = 0.688–0.995, *p* = 0.044) was significantly associated with the district having lower odds of having a wellness plan. Whereas, in the adjusted model, each one hundred students more per school (OR = 1.387; 95% CI = 1.110–1.733, *p* = 0.004) was significantly associated with the district having higher odds of having a SHAC.

## 4. Discussion

In South Texas, urban districts had, on average, two more LWP goals than rural districts, including a statistically higher number of nutrition education and other school-based activity goals than rural districts in both the unadjusted and adjusted models. Nutrition education goals include (a) promoting healthy nutrition messages in cafeterias, classrooms, and other appropriate settings; (b) sharing educational nutrition information with families and the public; and (c) ensuring that food and beverage advertisements contain only products that meet the federal guidelines for competitive foods. However, the inclusion of sharing educational nutrition information with families and the public was the only goal included less for rural vs. urban districts (72.0% vs. 86.9%). Two previously cited barriers to conducting nutrition education in rural settings include a lack of nutrition infrastructure to reinforce messages and transportation barriers [37]. However, it is unclear if those same barriers are affecting the goals rural districts choose to include in their LWPs. More research is needed to understand these findings.

Urban districts also had a higher number of physical activity and nutrition promotion goals in the unadjusted models; however, once adjusting for other covariates, these analyses were no longer significant. Revenue per student was an important covariate, signifying that budgetary constraints may be driving the number of physical activity and nutrition promotion goals that districts can write into their LWPs. Previous research has shown that the cost is an important factor that affects the delivery of physical activity and nutrition programs in the school setting [38,39,40]. Thus, it is likely that schools are also considering the cost of delivering these programs when they select goals for their LWPs. Based on the literature and our findings, it is also likely that rural districts have fewer other types of resources (e.g., staff with specialized training in physical activity and nutrition) than urban districts, which reinforces them setting fewer goals and metrics to meet [38,39,40]. It should be noted that the districts setting fewer goals may not have worse health environments. Future research can help us to understand how the number of goals in a district’s wellness policy is related to school health environments and students’ health outcomes.

Similarly, there was an urban–rural difference in the availability of wellness plans and SHACs in the unadjusted models. However, this difference no longer met the threshold for statistical significance when covariates were added to the model (the SHACs remained marginally significant; *p* < 0.10). Despite the change in *p*-values, which may be due, in part, to the relation of rurality with the number of students per school and/or district revenue, previous research suggests that stakeholders in rural schools see SHACs as a way to improve LWP implementation [33]. Additionally, LWP implementation is higher in districts with a SHAC, as they provide organizational support for developing, implementing, evaluating, and monitoring LWPs [40]. In Texas, all districts are required to have a SHAC. However, only 43.3% of rural districts had a SHAC on their website compared to 79.2% of urban districts, suggesting that more resources may be needed to develop and maintain effective SHACs in rural areas. Developing implementation support strategies that better utilize the WSCC model (e.g., community involvement and family engagement), such as starting a coalition, creating local champions, providing technical assistance, or accessing new funding, may increase the prevalence of SHACs in rural districts, [27,35] and ultimately, their impact on nutrition education and promotion.

In general, it is important to note that rurality is positively correlated with revenue per student (r = 0.320, *p* < 0.001) and negatively correlated with students per school (r = 0.611, *p* < 0.001), as well as several of the other covariates that we considered. When these covariates were included in the adjusted models, several of the statistically significant bivariate relationships were no longer statistically significant. Given that these characteristics often define rural school districts and are co-linear with rurality, it is unclear if these characteristics or other characteristics of rurality are affecting LWPs. Future work should aim to understand the specific demographic characteristics of rural districts that, independently or in combination, affect the development and implementation of LWPs.

### 4.1. Implications for School Health Policy, Practice, and Equity

Based on our findings, it is clear that rural school districts have fewer LWP goals than urban districts, which highlights a need for rural districts to prioritize the selection of goals that have the strongest impact on student health outcomes. In particular, more research studies that identify how LWP goals are related to the school physical activity and nutrition environments and student health behaviors are needed. Furthermore, tools to disseminate this information can help school districts make evidence-based decisions about which goals to include in their LWPs. Non-profit agencies, such as Action for Healthy Kids or Alliance for a Healthier Generation, play an important role in the information dissemination process, and they may represent one avenue to share information with school districts once evidence-based policies are identified.

More resources are also needed to create effective SHACs that can help develop and implement LWPs in rural areas. At the state level, agencies like the Texas Association of School Boards or the Texas Education Agency may need to develop resources for school districts to help facilitate the difficult process of creating SHACs in rural settings. They may also be able to develop resources for SHACs that facilitate the development of high-quality LWPs and LWP implementation processes. At more local levels, additional efforts from other community stakeholders (e.g., parent–teacher associations, school nurses, local community organizations, and universities) may be needed to supplement the role that the SHAC plays in ensuring school health within rural communities.

### 4.2. Strengths and Limitations

One strength of our analysis is that we included two samples from different regions of Texas. This variability in our data helped to ensure that the urban–rural differences we uncovered in LWP-related outcomes were not a regional phenomenon. Also, our data were derived from public sources, which allows others to replicate our results and provides an avenue to continuously monitor LWP-related outcomes. 

Despite the benefits of using publicly available sources, one limitation is that these sources may not reflect actual health practices in the district and instead may reflect compliance or resources to comply with government mandates to make this data publicly available. For example, schools with fewer resources may update their websites less frequently than schools with more resources. Government organizations, such as the Texas Education Agency, Texas Association of School Boards, or other non-profits (e.g., Action for Healthy Kids) could be potential avenues to provide support, formally or informally, to schools to ensure that LWPs are up to date and accurately reflect school health environments. Given that LWPs and website audits were collected from different districts, we could not assess if LWP supports were related to the number of goals in the LWPs. Future work should examine the processes by which LWP supports, such as SHAC, affect the number of goals in LWPs. Finally, there were demographic differences between the two regions included in this study.

## 5. Conclusions

Important urban–rural differences exist in Texas LWPs and LWP implementation environments. However, urban–rural differences in LWP implementation environments were not as strong and may be related to key district demographic characteristics. More research is needed to determine how the content of LWPs relates to LWP implementation processes. The results collected in this study help to identify opportunities to tailor and modify LWPs and LWP implementation processes to close current LWP gaps between rural and urban districts.

## Figures and Tables

**Table 1:** Goals listed in the Texas Local Wellness Policy template.

**Nutrition Promotion (NP)**
*Goal NP1:* The District’s food service staff, teachers, and other District personnel shall consistently promote healthy nutrition messages in cafeterias, classrooms, and other appropriate settings. *Goal NP2:* The District shall share educational nutrition information with families and the general public to promote healthy nutrition choices and positively influence the health of students. *Goal NP3:* The District shall ensure that food and beverage advertisements accessible to students outside of school hours on District property contain only products that meet the federal guidelines for competitive foods.
**Nutrition Education (NE)**
*Goal NE1:* The District shall deliver nutrition education that fosters the adoption and maintenance of healthy eating behaviors. *Goal NE2:* The District shall make nutrition education a District-wide priority and shall integrate nutrition education into other areas of the curriculum, as appropriate. *Goal NE3:* The District shall provide professional development so that teachers and other staff responsible for the nutrition education program are adequately prepared to effectively deliver the program. *Goal NE4:* The District shall establish and maintain school gardens and farm-to-school programs.
**Physical Activity (PA)**
*Goal PA1:* The District shall provide an environment that fosters safe, enjoyable, and developmentally appropriate fitness activities for all students, including those who are not participating in physical education classes or competitive sports. *Goal PA2:* The District shall provide appropriate staff development and encourage teachers to integrate physical activity into the academic curriculum where appropriate. *Goal PA3:* The District shall make appropriate before-school and after-school physical activity programs available and shall encourage students to participate. *Goal PA4:* The District shall make appropriate training and other activities available to District employees in order to promote enjoyable, lifelong physical activity for District employees and students. *Goal PA5:* The District shall encourage parents to support their children’s participation, to be active role models, and to include physical activity in family events. *Goal PA6:* The District shall encourage students, parents, staff, and community members to use the District’s recreational facilities, such as tracks, playgrounds, and the like, that are available outside of the school day.
**Other School-Based Activities (OSA)**
*Goal OSA1:* The District shall allow sufficient time for students to eat meals in cafeteria facilities that are clean, safe, and comfortable. *Goal OSA2:* The District shall promote wellness for students and their families at suitable District and campus activities. *Goal OSA3:* The District shall promote employee wellness activities and involvement at suitable District and campus activities.

**Table 2:** Descriptive characteristics of the two samples.

Characteristic	South Texas (*n* = 95)	North Texas (*n* = 239)
Rural (%)	42.1%	37.7%
Students per School (*M ± SD*)	605.1 ± 258.0 *	428.8 ± 233.8 *
Schools per District (*M ± SD*)	16.6 ± 32.9	9.5 ± 21.2
Students per District (*M ± SD*)	13,582.9 ± 28,391.3 *	6109.7 ± 15064.1 *
Students per Teacher (*M ± SD*)	14.7 ± 1.8 *	13.0 ± 2.2 *
Revenue per Student (*M ± SD*)	11,541.3 ± 1681.0 *	12,788.7 ± 1995.1 *
Student Demographics		
% Economically Disadvantaged (*M ± SD*)	55.9 ± 17.0	54.1 ± 18.8
% Black/African American (*M ± SD*)	12.4 ± 11.2 *	8.0 ± 12.3 *
% Hispanic/Latino (*M ± SD*)	38.0 ± 18.1 *	25.1 ± 16.0 *
% White (*M ± SD*)	44.7 ± 23.4 *	60.9 ± 22.2 *
% Other (*M ± SD*)	4.9 ± 3.5 *	6.1 ± 5.6 *

* *p* < 0.05.

**Table 3:** Urban–rural differences in the number of LWP goals.

	Model 1		Model 2	
	B	*p*-Value	B	*p*-Value
**Nutrition Promotion Goal**				
Rural	−0.316 *	0.034 *	−0.265	0.139
*Covariates*				
Percent economically disadvantaged			0.005	0.288
Revenue per student			−0.064	0.170
Students per school			0.000	0.897
**Nutrition Education Goal**				
Rural	−0.536 *	0.006 *	−0.654 *	0.005 *
*Covariates*				
Percent economically disadvantaged			−0.010	0.099
Revenue per student			−0.107	0.072
Students per school			0.000	0.316
**Physical Activity Goal**				
Rural	−0.830 *	0.038 *	−0.687	0.150
*Covariates*				
Percent economically disadvantaged			0.003	0.836
Revenue per student			−0.246 *	0.048 *
Students per school			0.000	0.991
**Other School-Based Activity**				
Rural	−0.668*	<0.001 *	−0.675 *	0.001 *
*Covariates*				
Percent economically disadvantaged			−0.005	0.322
Revenue per student			−0.036	0.495
Students per school			0.000	0.969
**Total Number of Goals**				
Rural	−2.350 *	0.003 *	−2.281 *	0.014 *
*Covariates*				
Percent economically disadvantaged			−0.007	0.753
Revenue per student			−0.453	0.059
Students per school			0.000	0.786

* *p* < 0.05.

**Table 4:** Urban–rural differences in supports for implementation of LWPs.

	OR (95% CI)	*p*-Value	OR (95% CI)	*p*-Value
**Wellness Plan**				
Rural	0.520 (0.288–0.939) *	0.030 *	0.936 (0.396–2.211)	0.880
**Triennial Assessment**				
Rural	1.029 (0.554–1.915)	0.927	1.547 (0.603–3.969)	0.364
**School Health Advisory Council (SHAC)**				
Rural	0.201 (0.113–0.357) *	<0.001 *	0.461 (0.193–1.105)	0.083

* *p* < 0.05.

## Data Availability

The data presented in this study are available on request from the corresponding author. The data are not publicly available due to privacy.
